# Staged versus immediate complete revascularization for non-culprit arteries in acute myocardial infarction: a post-hoc analysis of FRAME-AMI

**DOI:** 10.3389/fcvm.2024.1475483

**Published:** 2024-12-12

**Authors:** Yongwhan Lim, Jaehyuk Jang, Seung Hun Lee, Joon Ho Ahn, Young Joon Hong, Youngkeun Ahn, Myung Ho Jeong, Chan Joon Kim, Joo-Yong Hahn, Joo Myung Lee, Keun Ho Park, Eun Ho Choo, Sung Gyun Ahn, Joon-Hyung Doh, Sang Yeub Lee, Sang Don Park, Hyun-Jong Lee, Min Gyu Kang, Yun-Kyeong Cho, Chang Wook Nam, Sung Hyun Bu, Min Chul Kim

**Affiliations:** ^1^ Department of Cardiology, Chonnam National University School of Medicine, Chonnam National University Hospital, Gwangju, Republic of Korea; ^2^ Division of Cardiology, Department of Internal Medicine, Uijeongbu St. Mary’s Hospital, College of Medicine, The Catholic University of Korea, Uijeongbu-si, Republic of Korea; ^3^ Heart Vascular Stroke Institute, Samsung Medical Center, Sungkyunkwan University School of Medicine, Seoul, Republic of Korea; ^4^ Department of Cardiology, Chosun University Hospital, University of Chosun College of Medicine, Gwangju, Republic of Korea; ^5^ Department of Cardiology, Seoul St. Mary’s Hospital, The Catholic University of Korea, Seoul, Republic of Korea; ^6^ Department of Cardiology, Yonsei University Wonju College of Medicine, Wonju Severance Christian Hospital, Wonju, Republic of Korea; ^7^ Department of Medicine, Inje University Ilsan Paik Hospital, Goyang, Republic of Korea; ^8^ Department of Cardiology, Chung-Ang University College of Medicine, Chung-Ang University Gwangmyeong Hospital, Gwangmyeong, Republic of Korea; ^9^ Department of Cardiology, Inha University Hospital, Incheon, Republic of Korea; ^10^ Department of Cardiology, Sejong General Hospital, Bucheon, Republic of Korea; ^11^ Department of Cardiology, Gyeongsang National University School of Medicine, Gyeongsang National University Hospital, Jinju, Republic of Korea; ^12^ Department of Cardiology, Keimyung University Dongsan Medical Center, Daegu, Republic of Korea

**Keywords:** acute myocardial infarction, multivessel disease, staged complete revascularization, immediate complete revascularization, FRAME-AMI

## Abstract

**Background and objectives:**

The optimal timing for complete revascularization (CR) in patients with acute myocardial infarction (AMI) and multivessel disease (MVD) remain uncertain.

**Methods:**

This post-hoc analysis of the FRAME-AMI trial included AMI patients with MVD (*n* = 549). They were classified into immediate (*n* = 329) and staged CR (*n* = 220) groups. All percutaneous coronary interventions were performed during inex hospitalization. The primary endpoint was a composite of all-cause death, acute myocardial infarction, and repeated revascularization. Secondary endpoints included each component of the primary endpoint. Additional comparisons for the outcomes in ST-segment elevation myocardial infarction (STEMI) and non-STEMI (NSTEMI) were also performed.

**Results:**

The incidence of the primary endpoint was not significantly different in any of the AMI patients [12.7% [immediate CR] vs. 17.4% [staged CR], *p* = 0.905, adjusted hazard ratio [HR] of staged CR = 0.81, 95% confidence interval = 0.43–1.53, *p* = 0.528]. Other secondary endpoints were also not significantly different. Analyses of STEMI and Neither the primary or secondary endpoints of NSTEMI patients were significantly different.

**Conclusions:**

In this post-hoc analysis of the FRAME-AMI trial, no significant difference in clinical outcomes was observed between the immediate and staged CR strategies for AMI with MVD and the subgroups, such as STEMI or NSTEMI. However, the results should be interpreted carefully because of the many limitations, including a limited sample size and a lack of statistical power.

**Trial Registration:** FRAME-AMI clinicaltrials.gov, identifier (NCT02715518).

## Introduction

Multivessel disease (MVD) is present in 40%–50% of patients with acute myocardial infarction (AMI) (1–4). It is generally accepted that the prognosis of AMI with MVD is worse than that of its counterparts (1, 2), necessitating extensive research to optimize revascularization strategies for non-culprit arteries to improve patient outcomes.

Prior studies have consistently demonstrated that complete revascularization (CR) with percutaneous coronary intervention (PCI) for AMI with MVD yields better clinical outcomes than culprit-only PCI (4–9). In light of these findings, contemporary guidelines now advocate CR for patients with AMI and MVD (10, 11).

However, compared to the established benefit of CR in AMI with MVD, the optimal timing of PCI for non-culprit arteries in hemodynamically stable patients remains unclear. Despite recent guidelines recommending staged complete revascularization and advocating for selective immediate complete revascularization in AMI with MVD (10, 11), the supporting evidence for these recommendations is limited. Given the currently limited data from dedicated studies on this topic (12, 13), the search for further evidence to define the optimal timing of PCI for non-culprit arteries continues to be of value.

In this study, we present a post-hoc analysis of the fractional flow reserve (FFR) vs. angiography-guided strategy for the management of non-infarction related artery stenosis in patients with AMI from the FRAME-AMI trial. Our aim was to compare strategies with differing timings of PCI for non-culprit arteries—staged vs. immediate CR in AMI with MVD—using data derived from the FRAME-AMI trial. In addition, applying the FFR in PCI for non-culprit arteries to vary the timing of CR was also examined.

## Method

### Study population and treatment

The study protocol was approved by the ethics committees of the participating centers (IRB no: CNUH-2018-143) and adhered to the principles of the Declaration of Helsinki. All patients provided written informed consent before inclusion in the registry.

The FRAME-AMI trial design and its principal results have been discussed in detail previously, specifically in the appendix of the primary publication (14).

Briefly, the FRAME-AMI trial was an open-label, multicenter, randomized study that contrasted the outcomes of FFR-guided vs. angiography-guided PCI for non-culprit arteries in AMI with MVD. The culprit artery was defined as the artery related to AMI, and it was determined by the operator based on angiography and/or other modalities, including electrocardiogram, echocardiography, or intravascular imaging, if indicated. Other coronary arteries were considered non-culprit arteries. MVD was defined as stenosis > 50% in a non-culprit vessel with a diameter ≥ 2.0 mm, as visually estimated. The study included patients aged over 18 years diagnosed with AMI. Primary PCI for the culprit artery was undertaken within 12 h of symptom onset in cases of ST elevation myocardial infarction (STEMI), and within 72 h in non-ST elevation myocardial infarction (NSTEMI) cases. The trial protocol was approved by the institutional review board at each participating site.

Exclusion criteria included cardiogenic shock at presentation, unprotected left main coronary artery disease (stenosis > 50% by visual estimation), non-culprit arteries not amenable for PCI, severe stenosis with thrombolysis in myocardial infarction (TIMI) flow II or less, or chronic total occlusion. Further details of inclusion and exclusion criteria have been previously described (14).

Following the culprit artery PCI, evaluation and PCI for non-culprit arteries were conducted either immediately or at a later stage, based on the operator's discretion. While the interval between index and staged PCI was not pre-determined, staged PCI for non-culprit arteries was carried out during the index hospitalization. CR was defined when all non-culprit arteries were treated with PCI according to predefined criteria based on FFR or angiographic guidance as described below.

In the FFR group, FFR was carried out in all non-culprit arteries with a lesion(s) presenting > 50% stenosis on visual estimation, and PCI for non-culprit arteries was performed only when the FFR result was 0.80 or lower. In the angiography group, lesions with a diameter stenosis > 50% were treated with PCI. All medical treatments adhered to the current guidelines (15), and dual antiplatelet treatment was maintained for a minimum of 12 months.

**Figure 1:**
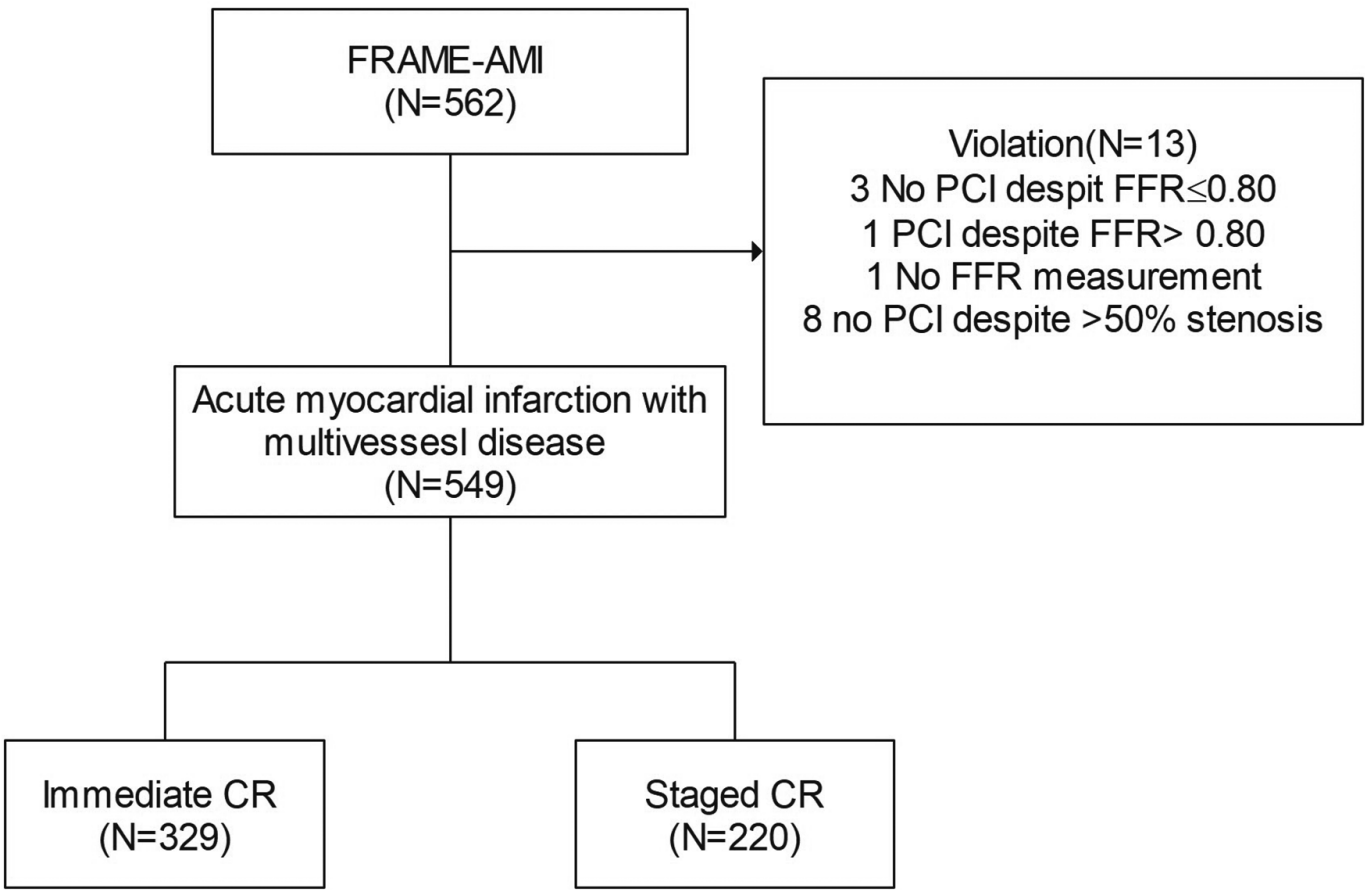
Flow for defining study subject. *Details of violation include 5 protocol violation in the FFR group (3 no PCI despite FFR < 0.80, 1 PCI despite FFR > 0.80, 1 no FFR measurement) and 8 protocol violation in the CAG group (no PCI for non-culprit). AMI, acute myocardial infarction; CR, complete revascularization.

This study's objective was to assess the impact of the timing of PCI for non-culprit arteries in AMI with MVD. To this end, we conducted a post-hoc analysis of the FRAME-AMI trial. Participants were categorized into immediate and staged CR groups based on the timing of evaluation and PCI for non-culprit arteries. All staged PCIs were performed during the index hospitalization. The flow of analyses is depicted in Figure 1. After excluding patients treated for non-culprit arteries in violation of the treatment protocol, comparisons were made for all AMI patients and separately for STEMI and NSTEMI cohorts.

### Clinical endpoints

The primary endpoint of this study was a composite of all-cause death, myocardial infarction (MI), or any repeat revascularization. Secondary endpoints encompassed each component of the primary endpoint, cerebrovascular accident (CVA), definitive or probable stent thrombosis, and contrast-induced nephropathy (CIN). The definition of death adhered to the guidelines set by the Academic Research Consortium (16). All deaths were presumed to be cardiac unless a distinct non-cardiac cause could be established. MI was defined in accordance with the third universal definition (17), which includes recurrent or procedural-related MI and thus encompasses periprocedural MI. The definition and classification of stent thrombosis adhered to the Academic Research Consortium's guidelines (16). More detailed definitions of clinical outcomes have been previously described in the appendix of the main paper (14).

### Statistical analysis

Categorical variables were analyzed using the chi-square or Fisher's exact tests. Continuous variables are reported as the mean ± SD or median with interquartile range and were analyzed using the Student's *t*-test or the Wilcoxon rank-sum test. A two-tailed *p*-value less than 0.05 was deemed to indicate statistical significance.

Kaplan–Meier analyses were conducted to compare the primary and secondary endpoints in groups with varying treatment strategies. The log-rank test was used to assess differences in survival between the groups.

Cox-proportional hazard regression models were employed to ascertain whether staged or immediate CR constitutes an independent predictor of clinical outcomes. Variables deemed significant in the univariable analysis (*p* < 0.1) or those with clinical significance for each outcome were included in the multivariable analysis. Multivariable Cox-proportional hazard models were prepared with propensity score (PS)-based overlap weighting as described below.

PSs were calculated to mitigate the confounding effects of variable differences in the variable distributions between the two groups and overlap weighting (OW) (18, 19) was applied. PSs were calculated for the revascularization strategies in the analyses (Supplementary Figures S1–S3) using the PSweight package. Several variables were adjusted for the timing of CR including age, sex, hypertension, systolic blood pressure (SBP), diastolic blood pressure, diabetes mellitus (DM), current smoking, door to balloon time, three-vessel disease, left main disease, location of the culprit lesion, transradial approach, use of a glycoprotein IIb-IIIA inhibitor, direct stenting for the culprit lesion, image-guided PCI for culprit vessels, creatinine, total numbers of culprit lesion(s) and non-culprit lesions, left ventricular ejection fraction, FFR guidance for non-culprit lesion, and STEMI in cases of all AMI. Histograms for PS and plots for PS density with weighting are provided for AMI, STEMI, and NSTEMI (Supplementary Figures S4–S6, respectively). Furthermore, subgroup analysis for the primary endpoint was performed for major clinically significant variables.

All statistical analyses were conducted using the R statistical package (version 4.2.0; R Foundation for Statistical Computing, Vienna, Austria).

## Results

Of the 562 patients with AMI and MVD who underwent PCI between August 2016 and December 2020, a total of 549 patients were analyzed after excluding those who violated the treatment protocol for their non-culprit artery.

In this analysis of patients with AMI and MVD, 329 patients (60.0%) underwent immediate complete revascularization, and 220 patients (40.0%) underwent staged complete revascularization. The median follow-up duration was 3.48 years (interquartile range 2.68 to 4.08 years).

### Baseline and procedural profiles in all AMI patients

The baseline clinical characteristics of the patients are presented in Table 1.

**Table 1:** Baseline characteristics in patients with all acute myocardial infarction.

	Immediate (*N* = 329)	Staged (*N* = 220)	*p*
Age	63.0 [56.0;71.0]	61.5 [55.0;72.0]	0.628
Male	267 (81.2%)	196 (89.1%)	0.017
BMI	24.6 [22.7;26.6]	24.8 [22.9;26.9]	0.659
SBP	130.0 [117.0;144.0]	125.0 [110.0;140.0]	0.009
DBP	80.0 [69.0;90.0]	77.0 [64.0;86.5]	0.056
HR	76.0 [66.0;85.0]	75.0 [62.0;86.0]	0.308
Hypertension	185 (56.2%)	111 (50.5%)	0.214
DM	120 (36.5%)	58 (26.4%)	0.017
Hyperlipidemia	135 (41.0%)	85 (38.6%)	0.636
Current smoker	107 (32.5%)	84 (38.2%)	0.203
Family history of CAD	26 (7.9%)	13 (5.9%)	0.471
CKD	12 (3.6%)	4 (1.8%)	0.322
Previous CVA	16 (4.9%)	8 (3.6%)	0.634
Previous MI	9 (2.7%)	4 (1.8%)	0.684
Previous PCI	24 (7.3%)	12 (5.5%)	0.498
Previous CHF	1 (0.3%)	0 (0.0%)	1
Peripheral vascular disease	5 (1.5%)	1 (0.5%)	0.449
Hemoglobin	14.4 [13.1;15.6]	14.4 [13.1;15.6]	0.707
Creatinine	0.9 [ 0.8; 1.1]	1.0 [ 0.8; 1.1]	0.028
HDL	41.0 [36.0;49.0]	41.0 [34.0;48.0]	0.351
LDL	122.0 [90.0;146.5]	116.5 [87.0;141.0]	0.397
HbA1C	6.1 [ 5.7; 7.0]	5.9 [ 5.6; 6.5]	0.01
LVEF	54.0 [47.0;62.0]	53.0 [45.9;58.4]	0.016
Discharge medication			
Aspirin	326 (99.1%)	217 (98.6%)	0.936
Warfarin or NOAC	9 (2.7%)	9 (4.1%)	0.529
Clopidogrel	90 (27.4%)	65 (29.5%)	0.644
Ticagrelor	146 (44.4%)	104 (47.3%)	0.562
Prasugrel	90 (27.4%)	49 (22.3%)	0.214
Statin	318 (96.7%)	215 (97.7%)	0.637
Beta blocker	256 (77.8%)	166 (75.5%)	0.59
ACE inhibitor or ARB	230 (69.9%)	145 (65.9%)	0.372
CCB	62 (18.8%)	28 (12.7%)	0.075

Values are mean ± SD or median [25 percentile, 75 percentiles] according to distribution.

ACE, angiotensin converting enzyme; ARB, angiotensin receptor blocker; BNP, brain natriuretic peptide; BMI, body mass index; CABG, coronary artery bypass graft; CAD, coronary artery disease; CCB, calcium channel blocker; CHF, congestive heart failure; CKD, chronic kidney disease; CVA, cerebrovascular accident; DBP, diastolic blood pressure; DM, diabetes mellitus; ESRD, end-stage renal disease; HbA1C, hemoglobin A1C; HDL-C, high-density lipoprotein cholesterol; HR, heart rate; LDL-C, low-density lipoprotein cholesterol; LVEF, left ventricular ejection fraction; MI, myocardial infarction; NOAC, new oral anticoagulant; PCI, percutaneous coronary intervention.

Several statistically significant differences were observed between the groups. The immediate CR group had a lower proportion of males, with higher SBP, more frequent DM with higher hemoglobin A1C, lower creatinine, and higher left ventricular ejection fraction.

The procedural profiles are compared in Table 2. Immediate CR was less frequently performed in patients with STEMI (33.1% vs. 66.8%, *p* < 0.001). The distribution of culprit vessels also differed (*p* < 0.001), with a higher proportion of left anterior descending (LAD) (40.4% vs. 26.4%) and a lower proportion of right coronary artery (RCA) (33.4% vs. 54.5%), and less frequent three-vessel disease (31.6% vs. 48.6%, *p* < 0.001) in the immediate CR group. Pre-PCI diameter of the stenosis and the FFR value for non-culprit arteries were comparable between the two groups, but the total length of the non-culprit lesions was longer in immediate CR.

**Table 2:** Procedural profiles and in-hospital complications in all AMI patients.

	Immediate (*N* = 329)	Staged (*N* = 220)	*p*
STEMI	109 (33.1%)	147 (66.8%)	<0.001
DBT (min)	270.5 [78.5;1049.5]	80.0 [59.0;173.0]	<0.001
Culprit vessel			<0.001
LAD	133 (40.4%)	58 (26.4%)	
LCX	86 (26.1%)	42 (19.1%)	
RCA	110 (33.4%)	120 (54.5%)	
3-vessel disease	104 (31.6%)	107 (48.6%)	<0.001
Left main disease	6 (1.8%)	11 (5.0%)	0.064
Culprit lesion No.	1.0 [1.0; 1.0]	1.0 [1.0; 1.0]	0.087
Non-culprit lesion No.	1.0 [1.0; 1.0]	1.0 [1.0; 2.0]	<0.001
Transfemoral	32 (9.7%)	56 (25.5%)	<0.001
Stent insertion for culprit lesion	322 (98.2%)	218 (99.1%)	0.24
Gp IIb IIIA inhibitor	50 (15.2%)	53 (24.1%)	0.012
Direct stenting for culprit artery	37 (11.2%)	11 (5.0%)	0.017
Image guided PCI for culprit artery	82 (24.9%)	36 (16.4%)	0.022
Culprit total No. of stents	1.2 ± 0.5	1.2 ± 0.5	0.743
Culprit-mean stent size	3.1 ± 0.5	3.2 ± 0.5	0.1
Culprit-mean stent length	28.2 ± 7.1	28.9 ± 7.5	0.214
Culprit-total stent length	35.5 ± 17.4	36.0 ± 16.4	0.758
Culprit-procedural success	328 (99.7%)	220 (100.0%)	1
FFR guided	170 (51.7%)	109 (49.5%)	0.688
Any PCI for non-culprit artery(s)	265 (80.5%)	185 (84.1%)	0.345
Stent insertion for non-culprit	256 (96.6%)	175 (94.6%)	0.356
Image guided PCI for non-culprit^a^	66 (24.9%)	68 (36.8%)	0.009
Non-culprit maximal diameter stenosis (%)^a^	76.0 ± 11.4	77.1 ± 11.5	0.218
Non-culprit total lesion length (mm)^a^	21.6 ± 11.7	24.3 ± 12.1	0.007
Non-culprit FFR before PCI	0.70 ± 0.1	0.70 ± 0.1	0.827
Non-culprit lesion diameter stenosis in QCA– no./total no. (%)			
50%–70%	182/425 (42.9%)	110/327 (34.9%)	0.016
70%–90%	196/425 (46.2%)	176/327 (54.3%)	0.034
>90%	46/425 (10.8%)	38/327 (11.7%)	0.794
Non-culprit total No. of stents	1.0 ± 0.8	1.3 ± 0.9	<0.001
Non-culprit-mean stent size^a^	3.0 ± 0.5	3.0 ± 0.5	0.672
Non-culprit-total stent length^a^	34.8 ± 20.0	43.8 ± 21.3	<0.001
Non-culpirt-procedural success^a^	265 (100.0%)	185 (100.0%)	NA
Total No. of stents	2.3 ± 0.9	2.5 ± 1.1	0.001
Hospital stays	3.0 [2.0, 4.0]	3.0 [1.5, 5.0]	0.197
In-hospital complications			
Any complications	12 (3.6%)	14 (6.4%)	0.206
CHF	3 (0.9%)	1 (0.5%)	0.916
Emergent PCI	0 (0%)	0 (0%)	NA
Emergent CABG	0 (0%)	0 (0%)	NA
Cardiogenic shock	1 (0.3%)	6 (2.7%)	0.036
Contrast reaction	1 (0.3%)	0 (0.0%)	1
Cardiac tamponade	0 (0.0%)	0 (0.0%)	NA
Bleeding at access site	2 (0.6%)	2 (0.9%)	1
Access site occlusion	0 (0.0%)	1 (0.5%)	0.839
Access site dissection	0 (0.0%)	0 (0.0%)	NA
Access site AV fistula	0 (0.0%)	0 (0.0%)	NA
Peripheral embolization	0 (0.0%)	1 (0.5%)	0.839
Pseudoaneurysm	0 (0.0%)	0 (0.0%)	NA
Cardiac arrest	0 (0.0%)	2 (0.9%)	0.313
CIN	1 (0.3%)	1 (0.5%)	1

Values are mean ± SD or median [25 percentile, 75 percentiles] according to distribution.

AMI, acute myocardial infarction; AV, arteriovenous; CABG, coronary artery bypass graft; CHF, congestive heart failure; CIN, contrast induced nephropathy; DBT, door to balloon time; FFR, fractional flow reserve; LAD, left anterior descending; LCX, left circumflex artery; NA, not available; PCI, percutaneous coronary intervention.

^a^ Patients who did not have PCI for non-culprit artery(s) were excluded.

Procedural differences during PCI between the two groups were noted. The immediate CR group showed a lower rate of transfemoral approach (9.7% vs. 25.5%, *p* < 0.001) and use of glycoprotein IIb-IIIa inhibitors (15.2% vs. 24.1%, *p* = 0.012). Image-guided PCI for the culprit artery was more frequent in the immediate CR group (24.9% vs. 16.4%, *p* = 0.022). However, image guidance for PCI of non-culprit arteries was less frequently used in this group (24.9% vs. 36.8%, *p* = 0.009). The rate of FFR usage was statistically comparable in both groups.

Although mean FFR value for non-culprit artery was comparable (0.70 ± 0.1), non-culprit arteries with diameter stenosis ranged 70 to 90% was more prevalent in the staged CR group (46.2% vs. 54.3, *p* = 0.034).

The total number of stents used in the whole PCI procedure was lower in the immediate group (2.3 ± 0.9 vs. 2.5 ± 1.1, *p* = 0.001), with a shorter total length of stents (34.8 ± 20.0 mm vs. 43.8 ± 21.3 mm, *p* < 0.001), and a significantly lower number of stents for non-culprit arteries (1.0 ± 0.8 vs. 1.3 ± 0.9, *p* < 0.001) in the immediate CR group.

The length of hospital stay was not statistically different (3.0 days as median in both groups, *p* = 0.197). The total rate of in-hospital complications was comparable between groups (3.6% vs. 6.4%, *p* = 0.206), except for a lower rate of cardiogenic shock in the immediate CR group (0.3% vs. 2.7%, *p* = 0.036).

### Baseline and procedural profiles in STEMI and NSTEMI

The baseline clinical characteristics of the patients are presented in Supplementary Table S1. Patients with STEMI and MVD were less frequently treated with immediate CR than those with NSTEMI (42.5% vs. 75%, *p* < 0.001). Baseline characteristics in both STEMI and NSTEMI groups were statistically similar except for a higher heart rate and DM prevalence in STEMI (34.9% vs. 21.1%, *p* = 0.021), and higher SBP in NSTEMI patients treated with immediate CR.

Procedural profiles are compared in Supplementary Table S2. For STEMI patients, the door-to-balloon time (DBT) for a culprit artery was comparable between the two groups (median: 72.5 min vs. 68.0 min, *p* = 0.113). The distribution of the culprit vessel significantly differed (*p* = 0.002), with a higher proportion of LAD (50.5% vs. 28.6%) and lower proportion of right coronary artery (RCA) (37.6% vs. 56.5%) in the immediate CR group. The transfemoral approach was less frequently used (11.9% vs. 25.9%, *p* = 0.009) during immediate CR. In contrast to the similar usage rate of imaging devices for the culprit artery, imaging guidance was less frequently used for non-culprit arteries in the immediate CR group (23.2% vs. 38.1%, *p* = 0.027). Additionally, a lower number (1.1 ± 0.6 vs. 1.3 ± 0.8, *p* = 0.023) and shorter total length of stents (32.3 ± 15.0 mm vs. 40.8 ± 20.9 mm, *p* = 0.001) were used for non-culprit arteries in immediate CR. The incidence of in-hospital complications was comparable.

In NSTEMI patients, the DBT was longer in the immediate CR group (median: 625.5 min vs. 350.0 min, *p* = 0.001). There was also a difference in the distribution of culprit vessels (*p* = 0.009) between the two groups; similar to STEMI, LAD was more frequent (35.5% vs. 21.9%) and RCA was less common (31.5% vs. 50.7%) in the immediate CR group. The transfemoral approach was also less frequently used in immediate multivessel PCI (8.6% vs. 24.7%, *p* = 0.001).

Fewer stents were used (1.0 ± 0.8 vs. 1.4 ± 1.0, *p* = 0.001) with a shorter total stent length (36.2 ± 22.3 mm vs. 50.1 ± 20.9 mm, *p* < 0.001) for non-culprit arteries during immediate CR. The incidence of in-hospital complications, including CIN, was comparable.

### Clinical outcomes and survival analysis

**Figure 2:**
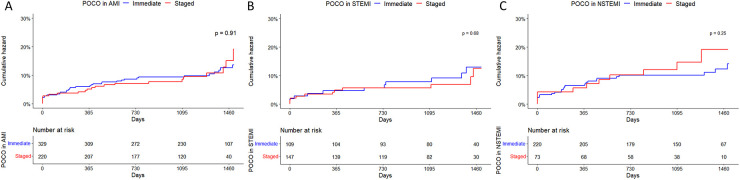
Kaplan-Meier curves of a composite outcome in patients with AMI **(A)**, STEMI **(B)** and NSTEMI **(C)** with multivessel disease treated with immediate or staged PCI strategy for non-culprit artery(s). AMI, acute myocardial infarction; CI, confidence interval; NSTEMI, non-ST segment elevation myocardial infarction; HR, hazard ratio; POCO, patient-oriented composite outcome; STEMI, ST segment elevation myocardial infarction.

Clinical outcomes are detailed in Table 3. The results of Kaplan-Meier analysis for the primary endpoints in AMI, STEMI, and NSTEMI are provided in Figure 2.

**Table 3:** Outcomes in AMI, STEMI and NSTEMI.

All AMI (*N* = 549)					
	Immediate (*N* = 329)	Staged (*N* = 220)	*p* ^c^	^b^Adjusted HR of staged CR (95% CI)	*p* value of HR
Composite outcome^a^	35 (12.7%)	22 (17.4%)	0.905	0.81 (0.43–1.53)	0.528
All-cause death	13 (4.4%)	8 (9.7%)	0.904	1.01 (0.98–1.03)	0.46
Cardiac death	11 (3.8%)	7 (9.3%)	0.815		
Any MI	18 (6.3%)	9 (4.2%)	0.518	1.01 (0.95–1.01)	0.28
Periprocedural MI	7 (2.1%)	6 (2.7%)	0.651		
Any repeated revascularization	17 (7.0%)	9 (6.3%)	0.766	1.00 (0.96–1.02)	0.55
Culprit artery	7 (3.3%)	5 (4.4%)	0.685		
Non-culprit artery	13 (4.8%)	6 (3.2%)	0.534		
Any CVA	2 (0.6%)	4 (1.8%)	0.185		
Ischemic CVA	2 (0.6%)	4 (1.8%)	0.185		
Definitive or probable stent thrombosis	0 (0.0%)	1 (0.5%)	0.222		
CIN	1 (0.3%)	1 (0.5%)	0.775		
STEMI (*N* = 256)					
	Immediate (*N* = 109)	Staged (*N* = 147)	*p* ^c^	^b^Adjusted HR of staged CR (95% CI)	*p* value of HR
Composite outcome^a^	11 (12.1%)	11 (11.8%)	0.676	0.98 (0.93–1.03)	0.49
All-cause death	1 (1.2%)	4 (5.2%)	0.204	1.01 (0.99–1.03)	0.17
Cardiac death	1 (1.2%)	4 (5.2%)	0.204		
Any MI	6 (5.6%)	4 (2.7%)	0.273	0.98 (0.94–1.02)	0.34
Periprocedural MI	5 (1.8%)	3 (2.0%)	0.908		
Any repeated revascularization	8 (9.1%)	4 (5%)	0.151	0.97 (0.93–1.01)	0.2
Culprit artery	3 (3.8%)	3 (4.3%)	0.891		
Non-culprit artery	6 (6.3%)	2 (1.4%)	0.081		
Any CVA	0 (0.0%)	2 (1.4%)	0.221		
Ischemic CVA	0 (0.0%)	2 (1.4%)	0.221		
Definitive or probable stent thrombosis	0 (0.0%)	0 (0.0%)	1		
CIN	0 (0.0%)	0 (0.0%)	1		
NSTEMI (*N* = 293)					
	Immediate (*N* = 220)	Staged (*N* = 73)	*p* ^c^	^b^Adjusted HR of staged CR (95% CI)	*p* value of HR
Composite outcome^a^	24 (13.2%)	11 (29.2%)	0.252	0.99 (0.91–1.07)	0.84
All-cause death	12 (6.0%)	4 (20.3%)	0.849	1.00 (0.94–1.08)	0.8
Cardiac death	10 (5.1%)	3 (19.2%)	0.959		
Any MI	12 (6.7%)	5 (7.2%)	0.615	0.97 (0.93–1.02)	0.37
Periprocedural MI	2 (2.3%)	3 (4.1%)	0.405		
Any repeated revascularization	9 (6.0%)	5 (8.4%)	0.264	0.99 (0.94–1.04)	0.79
Culprit artery	4 (3.1%)	2 (4.0%)	0.996		
Non-culprit artery	7 (4.0%)	4 (7.0%)	0.315		
Any CVA	2 (0.9%)	2 (2.7%)	0.248		
Ischemic CVA	2 (0.9%)	2 (2.7%)	0.248		
Definitive or probable stent thrombosis	0 (0.0%)	1 (1.4%)	0.085		
CIN	1 (0.5%)	1 (1.4%)	0.413		

^a^ Composite outcome = all-cause death + myocardial infarction (including periprocedural MI) + any repeated revascularization.

^b^ Overlap weighting adjusted multivariable analysis.

^c^ *p* by log-rank test.

CIN, contrast induced nephropathy; CR, complete revascularization; CVA, cerebrovascular accident; HR, hazard ratio; MI, myocardial infarction; NSTEMI, non-ST segment elevation myocardial infarction; RR, peated revascularization; ST, stent thrombosis; STEMI, ST segment elevation myocardial infarction.

Among all AMI patients, during the follow-up period (median 3.48 years, 1Q/3Q = 2.68/4.08 years), the primary endpoint occurred in 35 and 22 patients in the immediate and staged CR groups, respectively. All-cause death occurred in 13 and 8 patients in each group. Among them, 11 and 7 cardiac deaths occurred in each group. There was no in-hospital mortality in either group. No other outcomes were significantly different.

In patients with STEMI, composite outcomes occurred in 11 patients in each group and all-cause death occurred in 1 and 4 patients in each group. All mortalities were cardiac-related. In patients with NSTEMI, the primary endpoint occurred in 24 and 11 patients in each group (13.2% vs. 29.2%). All-cause death occurred in 12 and 4 patients in each group (6.0% vs. 20.3%). Other secondary endpoints did not significantly differ. The results of Kaplan-Meier analysis for secondary endpoints in AMI, STEMI, and NSTEMI are provided in Supplementary Figures S7–S9.

### Subgroup analysis

**Figure 3:**
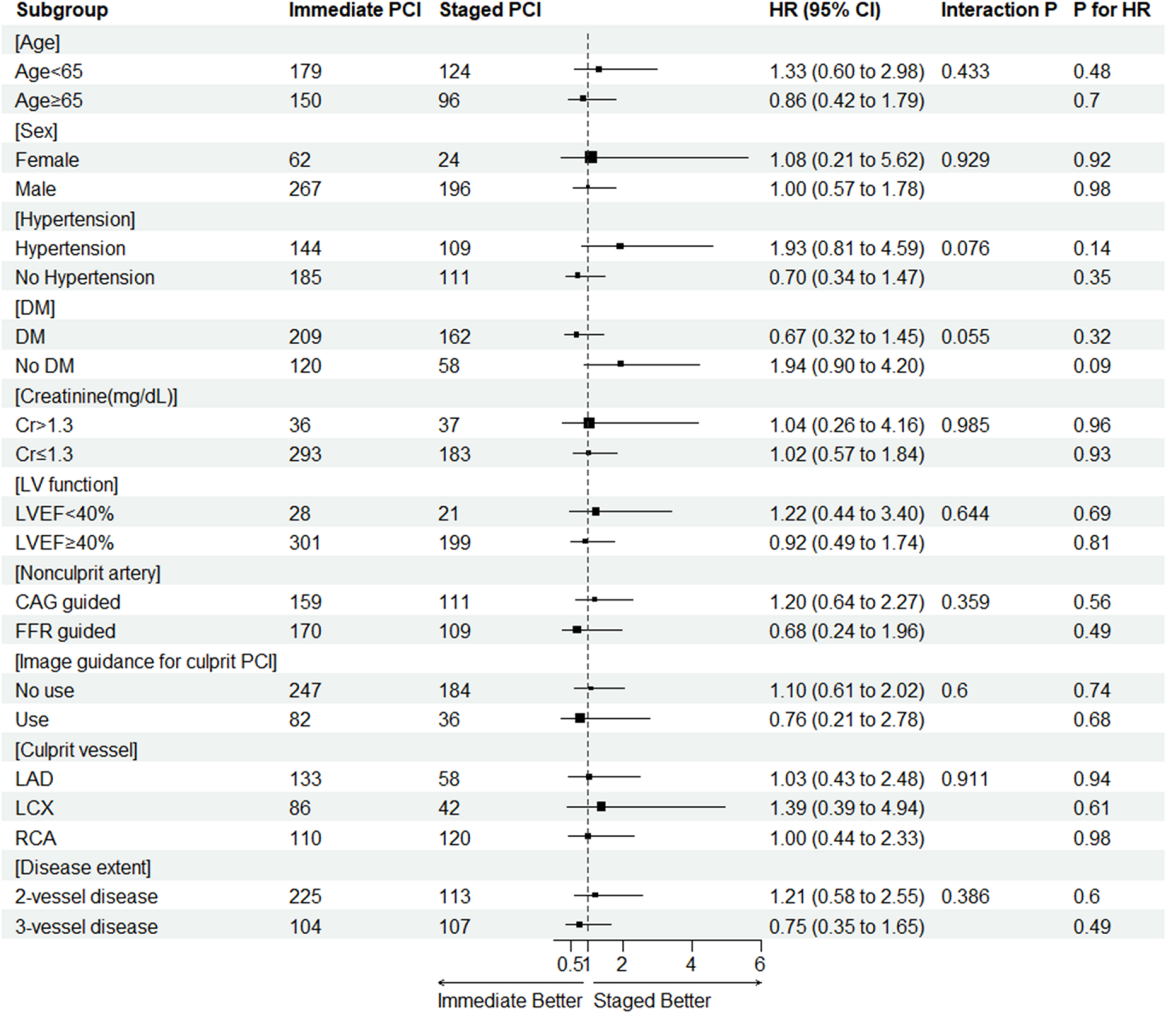
The result of subgroup analysis for the primary endpoint. CAG, coronary angiography; CI, confidence interval; Cr, creatinine (md/dl); DM, diabetes mellitus; EF, ejection fraction; FFR, fractional flow reserve; HR, hazard ratio; LAD, left anterior descending; LCX, left circumflex; LV, left ventricle; NSTEMI, non-ST segment elevation myocardial infarction; PCI, percutaneous coronary intervention; RCA, right coronary artery.

The results of the subgroup analysis for the primary endpoint are displayed in Figure 3. Because subgroups according to presentation (STEMI or NSTEMI) were analyzed in detail separately, the subgroups were not included in Figure 3 (interaction *p* = 0.281). There were no statistically significant differences in HR for the primary endpoint from either immediate or staged PCI for nonculprit arteries in all analyzed subgroups.

## Discussion

The current study draws upon data from the FRAME-AMI trial to compare clinical outcomes between two different revascularization strategies for non-culprit arteries in AMI with MVD: immediate vs. staged CR, followed by detailed subgroup evaluations of STEMI and NSTEMI patients.

Our findings can be summarized as follows:

First, during the FRAME-AMI trial, approximately 60% of patients with AMI and MVD underwent immediate PCI for non-culprit arteries. It is worth noting that immediate CR at the time of index PCI was less frequent in patients with STEMI and MVD compared to those with NSTEMI (42.5% vs. 75%, *p* < 0.001).

Second, our analysis revealed no statistically significant difference in both primary and secondary endpoints based on the timing of CR over a median follow-up period of 3.48 years in AMI, STEMI, and NSTEMI patients. These statistical similarities extended to potential periprocedural complications, including CIN, stroke, or cardiogenic shock or arrest.

Third, a subgroup analysis for the primary endpoint showed no difference in risk for the outcome between immediate and staged CR across all subgroups.

Despite the well-established benefits of CR in AMI with MVD (4–11), the timing of PCI for non-culprit arteries remains a contested point in practice. For instance, immediate CR raises concerns about potential additional complications for non-culprit arteries, such as CIN (20), or a deterioration in patient status, which might be challenging to manage in detail during an extended PCI. This is due to the additional immediate PCI for non-culprit arteries, which prolongs the procedure time and increases the contrast dose. Moreover, the extended procedural time related to additional procedures at the time of index PCI could disrupt the workflow of a catheterization laboratory, presenting a practical issue for immediate CR in some settings.

Compared to immediate CR, a staged complete revascularization strategy might also pose certain challenges such as the risk of periprocedural complications related to additional vascular access at the time of staged PCI. Moreover, uncertainty about the culprit artery(s), particularly in NSTEMI (21, 22), could complicate the index PCI in some instances, unlike the immediate complete revascularization strategy where the non-culprit artery could be evaluated and treated during the index PCI. Potential plaque instability and subsequent events (23) during the interval between index and staged PCI could be an additional concern. Furthermore, the potential for increased socioeconomic costs due to a staged procedure or prolonged duration of hospitalization might be a disadvantage of a staged CR strategy in real-world practice.

Considering these issues, evidence from a limited number of dedicated, prospective studies suggests that immediate CR could be selectively chosen with comparable outcomes. For instance, Gennaro et al. (13) compared outcomes in NSTEMI with MVD patients treated with immediate or staged CR performed during the index hospitalization (mean 4.76 days). They found a better composite outcome in the group treated with immediate PCI for non-culprit artery(s), mainly driven by a lower rate of one-year target vessel revascularization. The immediate PCI strategy was not associated with a higher risk of CIN and was linked to a more rapid decrease in troponin levels during the periprocedural period. Compared to the immediate CR group, staged CR was associated with an increase in troponin T levels 12 h after index PCI. Furthermore, additional vascular access during staged PCI was associated with a higher rate of minor bleeding, but not major bleeding.

The recently published results from the BIOVASC trial, which examined biodegradable polymer-coated stents in patients with acute coronary syndrome and multivessel disease, provided further insight into the optimal timing of PCI for non-culprit arteries in AMI with MVD. In this prospective multicenter trial, which compared immediate and staged CR in AMI with MVD, Roberto et al. demonstrated the non-inferiority of the immediate complete revascularization strategy for a 1-year composite outcome (12). Their analysis showed that staged complete revascularization was associated with a higher incidence of MI and unplanned ischemia-driven revascularization. In their study, staged PCI for non-culprit arteries was performed later during the index hospitalization or through re-admission within six weeks after the index procedure. Interestingly, 44.1% of MI occurred in the interval between the index and staged PCI, with 66% of these MIs associated with non-culprit arteries.

In comparison with the data from the BIOVASC trial, our analysis did not show a significant difference in MI or any repeated revascularization in either the culprit or non-culprit arteries between the immediate and staged complete revascularization groups. These findings could be attributed to the shorter interval between the index and staged PCI for the non-culprit artery in our study. While the interval between the index and staged PCI was not predetermined in the study protocol, all PCIs were performed during the index hospitalization in our study. The average hospital stay for staged CR was 3.0 days (1Q/3Q = 1.0/5.0 days), which is shorter than the median interval to staged PCI (15 days) in the BIOVASC trial. These differences may provide insights for determining the optimal interval between the index and staged PCI for non-culprit arteries, to reduce clinical events in future studies.

The recently published MULTISTARS AMI study (24), compared immediate CR with staged CR in hemodynamically stable STEMI patients with MVD, and also showed a higher risk of unplanned ischemia-driven revascularization in the staged CR arm. In that study, the interval between the index procedure and the staged procedure was longer than ours, 19–45 days in protocol (median 37 days), and more than half of the events occurred during the interval (23 of 39 patients). Based on these and our observations, we hypothesized that the interval between immediate and staged interventions could affect adverse events in a staged CR strategy, so further study about the optimal interval between the index and staged interventions in patients with AMI and MVD is suggested.

In addition to the difference in the interval to staged PCI between studies, differences in the definition of CR caused by measures to determine the significance of non-culprit arteries should also be discussed. Although the definition of CR to treat non-culprit arteries with significance is basically the same, the non-culprit artery was treated with PCI based on angiographic guidance in the BIOVASC and MULTISTARS AMI studies, and the decision for PCI was left to the operator's discretion in the BIOVASC trial. Physiological testing was not mandatory in less than 20% of the cases. Thus, our results should be interpreted differently from these two trials. PCI for non-culprit arteries was performed in 81.9% of cases in the FRAME-AMI trial, and less frequently in the FFR guidance group (64.1%). This means that about 20% of patients in our study were treated with functional CR in which the non-culprit artery with hyperemic FFR ≥ 0.80 was treated medically. Compared to our data, the investigator-reported CR rate was 96.1% in the BIOVASC trial. In addition, a significant proportion of non-culprit arteries in the FRAME-AMI trial (38.8%) had diameter stenoses ranging from 50 to 70%, which was not significant in the BIOVASC and MULTISTARS AMI trials. These lesions were evaluated and treated with PCI in the angiography-guided group. Differences in the mandatory use of FFR and the protocolized treatment and criteria for enrollment (>50% vs. 70%) of non-culprit arteries has led to differences in the definitions of CR between studies and should be considered for appropriate interpretation.

## Limitations

Several limitations in our study should be acknowledged. During the FRAME-AMI trial, patients were not randomly assigned to either of the CR strategies; the choice was determined by the operators’ decisions, which were likely to have been influenced by various clinical factors such as baseline renal function, the distribution of culprit and nonculprit arteries, the complexity of the nonculprit artery, disease extent, or the usage of imaging guidance for culprit artery PCI, as seen in our analysis. Although we attempted to reduce the impact of this patient profile disparity between the two revascularization strategies by applying propensity score-based methodology in the Cox proportional hazard model, the results should be interpreted with caution. Moreover, some of the variables such as the interval between the index and staged revascularization, procedural time, and contrast or radiation dose, were not collected and provided by the original study data.

Insufficient sample size and a lack of statistical power were important limitations. Because the original study was not constructed for this topic, the results of our analysis are inconclusive because of these limitations. Nevertheless, our analysis adds valuable insight about this topic because of some distinct features in the data, such as the mandatory use of physiological testing and protocolized PCI for nonculprit arteries according to the interval to staged PCI.

Finally, our results are not applicable to patients with cardiogenic shock or chronic total occlusion, as these populations were excluded in the original study.

In conclusion, our study demonstrated no significant differences in clinical outcomes between immediate and staged CR strategies for AMI with MVD and its subgroups, such as STEMI or NSTEMI, in the FRAME-AMI population. However, the results should be interpreted carefully because of limitations, including a limited sample size and a lack of statistical power.

## Data Availability

The raw data supporting the conclusions of this article will be made available by the authors, without undue reservation.

## References

[1] Park DW Clare RM Schulte PJ Pieper KS Shaw LK Califf RM Extent, location, and clinical significance of non-infarct-related coronary artery disease among patients with ST-elevation myocardial infarction. JAMA. (2014) 312(19):2019–27. 10.1001/jama.2014.15095 25399277

[2] Sorajja P Gersh BJ Cox DA McLaughlin MG Zimetbaum P Costantini C Impact of multivessel disease on reperfusion success and clinical outcomes in patients undergoing primary percutaneous coronary intervention for acute myocardial infarction. Eur Heart J. (2007) 28(14):1709–16. 10.1093/eurheartj/ehm184 17556348

[3] Baumann AAW Tavella R Air TM Mishra A Montarello NJ Arstall M Prevalence and real-world management of NSTEMI with multivessel disease. Cardiovasc Diagn Ther. (2022) 12(1):1–11. 10.21037/cdt-21-518 35282665 PMC8898694

[4] Rathod KS Koganti S Jain AK Astroulakis Z Lim P Rakhit R Complete versus culprit-only lesion intervention in patients with acute coronary syndromes. J Am Coll Cardiol. (2018) 72(17):1989–99. 10.1016/j.jacc.2018.07.089 30336821

[5] Engstrom T Kelbaek H Helqvist S Hofsten DE Klovgaard L Holmvang L Complete revascularisation versus treatment of the culprit lesion only in patients with ST-segment elevation myocardial infarction and multivessel disease (DANAMI-3-PRIMULTI): an open-label, randomised controlled trial. Lancet. (2015) 386(9994):665–71. 10.1016/S0140-6736(15)60648-1 26347918

[6] Gershlick AH Khan JN Kelly DJ Greenwood JP Sasikaran T Curzen N Randomized trial of complete versus lesion-only revascularization in patients undergoing primary percutaneous coronary intervention for STEMI and multivessel disease: the CvLPRIT trial. J Am Coll Cardiol. (2015) 65(10):963–72. 10.1016/j.jacc.2014.12.038 25766941 PMC4359051

[7] Smits PC Boxma-de Klerk BM. Fractional flow reserve-guided multivessel angioplasty in myocardial infarction. N Engl J Med. (2017) 377(4):397–8. 10.1056/NEJMc1706275 28745981

[8] Mehta SR Wood DA Storey RF Mehran R Bainey KR Nguyen H Complete revascularization with multivessel PCI for myocardial infarction. N Engl J Med. (2019) 381(15):1411–21. 10.1056/NEJMoa1907775 31475795

[9] Wald DS Morris JK Wald NJ Chase AJ Edwards RJ Hughes LO Randomized trial of preventive angioplasty in myocardial infarction. N Engl J Med. (2013) 369(12):1115–23. 10.1056/NEJMoa1305520 23991625

[10] Collet JP Thiele H Barbato E Barthelemy O Bauersachs J Bhatt DL 2020 ESC guidelines for the management of acute coronary syndromes in patients presenting without persistent ST-segment elevation. Eur Heart J. (2021) 42(14):1289–367. 10.1093/eurheartj/ehaa575 32860058

[11] Lawton JS Tamis-Holland JE Bangalore S Bates ER Beckie TM Bischoff JM 2021 ACC/AHA/SCAI guideline for coronary artery revascularization: a report of the American college of cardiology/American heart association joint committee on clinical practice guidelines. Circulation. (2022) 145(3):e18–114. 10.1161/CIR.0000000000001038 34882435

[12] Diletti R den Dekker WK Bennett J Schotborgh CE van der Schaaf R Sabate M Immediate versus staged complete revascularisation in patients presenting with acute coronary syndrome and multivessel coronary disease (BIOVASC): a prospective, open-label, non-inferiority, randomised trial. Lancet. (2023) 401(10383):1172–82. 10.1016/S0140-6736(23)00351-3 36889333

[13] Sardella G Lucisano L Garbo R Pennacchi M Cavallo E Stio RE Single-Staged compared with multi-staged PCI in multivessel NSTEMI patients: the SMILE trial. J Am Coll Cardiol. (2016) 67(3):264–72. 10.1016/j.jacc.2015.10.082 26796390

[14] Lee JM Kim HK Park KH Choo EH Kim CJ Lee SH Fractional flow reserve versus angiography-guided strategy in acute myocardial infarction with multivessel disease: a randomized trial. Eur Heart J. (2023) 44(6):473–84. 10.1093/eurheartj/ehac763 36540034

[15] Levine GN Bates ER Bittl JA Brindis RG Fihn SD Fleisher LA 2016 ACC/AHA guideline focused update on duration of dual antiplatelet therapy in patients with coronary artery disease: a report of the American college of cardiology/American heart association task force on clinical practice guidelines: an update of the 2011 ACCF/AHA/SCAI guideline for percutaneous coronary intervention, 2011 ACCF/AHA guideline for coronary artery bypass graft surgery, 2012 ACC/AHA/ACP/AATS/PCNA/SCAI/STS guideline for the diagnosis and management of patients with stable ischemic heart disease, 2013 ACCF/AHA guideline for the management of ST-elevation myocardial infarction, 2014 AHA/ACC guideline for the management of patients with non-ST-elevation acute coronary syndromes, and 2014 ACC/AHA guideline on perioperative cardiovascular evaluation and management of patients undergoing noncardiac surgery. Circulation. (2016) 134(10):e123–55. 10.1161/CIR.0000000000000404 27026020

[16] Garcia-Garcia HM McFadden EP Farb A Mehran R Stone GW Spertus J Standardized end point definitions for coronary intervention trials: the academic research consortium-2 consensus document. Eur Heart J. (2018) 39(23):2192–207. 10.1093/eurheartj/ehy223 29897428

[17] Thygesen K Alpert JS Jaffe AS Simoons ML Chaitman BR White HD Third universal definition of myocardial infarction. Glob Heart. (2012) 7(4):275–95. 10.1016/j.gheart.2012.08.001 25689940

[18] Li F Thomas LE Li F. Addressing extreme propensity scores via the overlap weights. Am J Epidemiol. (2019) 188(1):250–7. 10.1093/aje/kwy201 30189042

[19] Thomas LE Li F Pencina MJ. Overlap weighting: a propensity score method that mimics attributes of a randomized clinical trial. JAMA. (2020) 323(23):2417–8. 10.1001/jama.2020.7819 32369102

[20] Chen SL Zhang J Yei F Zhu Z Liu Z Lin S Clinical outcomes of contrast-induced nephropathy in patients undergoing percutaneous coronary intervention: a prospective, multicenter, randomized study to analyze the effect of hydration and acetylcysteine. Int J Cardiol. (2008) 126(3):407–13. 10.1016/j.ijcard.2007.05.004 17651830

[21] Kerensky RA Wade M Deedwania P Boden WE Pepine CJ, Veterans Affairs Non QWISi-HTI. Revisiting the culprit lesion in non-Q-wave myocardial infarction. Results from the VANQWISH trial angiographic core laboratory. J Am Coll Cardiol. (2002) 39(9):1456–63. 10.1016/S0735-1097(02)01770-9 11985907

[22] Balbi MM Scarparo P Tovar MN Masdjedi K Daemen J Den Dekker W Culprit lesion detection in patients presenting with non-ST elevation acute coronary syndrome and multivessel disease. Cardiovasc Revasc Med. (2022) 35:110–8. 10.1016/j.carrev.2021.03.019 33839051

[23] Goldstein JA Demetriou D Grines CL Pica M Shoukfeh M O'Neill WW. Multiple complex coronary plaques in patients with acute myocardial infarction. N Engl J Med. (2000) 343(13):915–22. 10.1056/NEJM200009283431303 11006367

[24] Stahli BE Varbella F Linke A Schwarz B Felix SB Seiffert M Timing of complete revascularization with multivessel PCI for myocardial infarction. N Engl J Med. (2023) 389(15):1368–79. 10.1056/NEJMoa2307823 37634190

