# Supplementary material for: Staged versus immediate complete revascularization for non-culprit arteries in acute myocardial infarction: a post-hoc analysis of FRAME-AMI
Source: Front Cardiovasc Med. 2024 Dec 12;11:1475483. doi: 10.3389/fcvm.2024.1475483 (PMC11669547; doi:10.3389/fcvm.2024.1475483)
Supplement: Supplementary file 1 [file Datasheet1.pdf]

Supplementary Material

Staged versus Immediate Complete Revascularization for Non-culprit Arteries in Acute Myocardial Infarction:

A Post-hoc Analysis of FRAME-AMI

On behalf of the FRAME-AMI investigators

Table of contents

Supplementary Tables

1. Baseline characteristics for varying complete revascularization timings (CR) in STEMI and NSTEMI
2. Hazard ratio of staged complete revascularization for outcomes, multivariable Cox-proportional Hazard Modelling, all patients with AMI
3. Supplementary Table 3. Results of Cox Proportional Hazard Modelling
4. Supplementary Table 4. Final models of OW-adjusted multivariable Cox proportional models for composite outcome

Supplementary Figures

1. Balancing using overlap weighting, all acute myocardial infarction (AMI)
2. Balancing using overlap weighting, ST segment elevation myocardial infarction (STEMI)
3. Balancing using overlap weighting, non-STEMI (NSTEMI)
4. Histogram for propensity score (A), and density plot describing overlap weighting in all patients (B, C)
5. Histogram for propensity score (A), and density plot describing overlap weighting in patients with ST-segment elevation myocardial infarction (B, C)
6. Histogram for propensity score (A), and density plot describing overlap weighting in patients with non-ST-segment elevation myocardial infarction (B, C)
7. Kaplan-Meier curve of all-cause death (A), myocardial infarction (MI) (B), and repeated revascularization (C) in patients with AMI with multivessel disease treated with immediate or staged PCI strategy for non-culprit artery(s).
8. Kaplan-Meier curve of all-cause death (A), MI (B), and repeated revascularization (C) in patients with STEMI with multivessel disease treated with immediate or staged PCI strategy for

non-culprit artery(s).

9. Kaplan-Meier curve of all-cause death (A), MI (B), and repeated revascularization (C) in pati ents with NSTEMI with multivessel disease treated with immediate or staged PCI strategy fo r non-culprit artery

Supplementary Table 1. Baseline characteristics in patients with STEMI and NSTEMI

	**STEMI (N=256)**			**NSTEMI (N=293)**		
	Immediate (N=109)	Staged (N=147)	P	Immediate (N=220)	Staged (N=73)	P
Age	63.0 [55.0;72.0]	62.0 [56.0;71.0]	0.858	63.0 [56.0;71.0]	61.0 [54.0;73.0]	0.556
Male	88 (80.7%)	131 (89.1%)	0.088	179 (81.4%)	65 (89.0%)	0.18
BMI	24.4 [22.2;26.4]	24.6 [23.0;26.9]	0.471	24.9 [22.9;26.6]	25.0 [22.6;26.6]	0.983
SBP	127.0 [113.0;147.0]	124.0 [106.0;142.0]	0.237	130.0 [120.0;143.0]	126.0 [112.0;140.0]	0.047
DBP	80.0 [67.0;90.0]	80.0 [64.0;90.0]	0.285	78.3 ± 13.2	74.9 ± 13.7	0.062
HR	78.4 ± 16.1	74.1 ± 17.2	0.043	75.0 [67.0;84.5]	76.0 [67.0;87.5]	0.452
Hypertension	62 (56.9%)	77 (52.4%)	0.557	123 (55.9%)	34 (46.6%)	0.211
DM	38 (34.9%)	31 (21.1%)	0.021	82 (37.3%)	27 (37.0%)	1
Hyperlipidemia	43 (39.4%)	58 (39.5%)	1	92 (41.8%)	27 (37.0%)	0.555
Current smoker	39 (35.8%)	57 (38.8%)	0.72	68 (30.9%)	27 (37.0%)	0.414

Family history of CAD	5 (4.6%)	8 (5.4%)	0.984	21 (9.5%)	5 (6.8%)	0.642
CKD	2 (1.8%)	2 (1.4%)	1	10 (4.5%)	2 (2.7%)	0.739
Previous CVA	4 (3.7%)	5 (3.4%)	1	12 (5.5%)	3 (4.1%)	0.884
Previous MI	2 (1.8%)	4 (2.7%)	0.964	7 (3.2%)	0 (0.0%)	0.271
Previous PCI	4 (3.7%)	11 (7.5%)	0.31	20 (9.1%)	1 (1.4%)	0.051
Previous CHF	0 (0.0%)	0 (0.0%)	1	1 (0.5%)	0 (0.0%)	1
Peripheral vascular disease	1 (0.9%)	1 (0.7%)	1	4 (1.8%)	0 (0.0%)	0.563
Hemoglobin	14.5 [13.4;15.8]	14.7 [13.2;15.6]	0.973	14.3 [12.9;15.5]	14.3 [12.8;15.4]	0.698
Creatinine	0.9 [0.8; 1.1]	1.0 [0.8; 1.1]	0.388	0.9 [0.8; 1.0]	0.9 [0.8; 1.2]	0.281
HDL	42.0 [36.0;50.0]	40.5 [34.0;48.0]	0.115	40.8 [35.5;48.5]	41.0 [37.0;48.0]	0.728
LDL	123.0 [97.0;154.0]	117.5 [94.5;142.0]	0.259	121.8 [86.0;142.0]	116.0 [73.0;136.0]	0.452
HbA1C	6.0 [5.7; 7.0]	5.9 [5.5; 6.5]	0.201	6.3 [5.7; 7.1]	5.8 [5.6; 6.6]	0.069
LVEF	50.0 [43.0;55.8]	52.0 [45.0;57.0]	0.225	57.0 [50.0;63.2]	55.0 [48.0;63.2]	0.162

Discharge medication						
Aspirin	108 (99.1%)	144 (98.0%)	0.836	218 (99.1%)	73 (100.0%)	1
Warfarin or NOAC	3 (2.8%)	6 (4.1%)	0.82	6 (2.7%)	3 (4.1%)	0.84
clopidogrel	28 (25.7%)	36 (24.5%)	0.942	62 (28.2%)	29 (39.7%)	0.089
Ticagrelor	53 (48.6%)	78 (53.1%)	0.565	93 (42.3%)	26 (35.6%)	0.387
Prasugrel	27 (24.8%)	32 (21.8%)	0.679	63 (28.6%)	17 (23.3%)	0.461
Statin	104 (95.4%)	143 (97.3%)	0.647	214 (97.3%)	72 (98.6%)	0.829
Beta blocker	86 (78.9%)	118 (80.3%)	0.91	170 (77.3%)	48 (65.8%)	0.072
ACE inhibitor or ARB	75 (68.8%)	97 (66.0%)	0.733	155 (70.5%)	48 (65.8%)	0.543
CCB	14 (12.8%)	18 (12.2%)	1	48 (21.8%)	10 (13.7%)	0.181

Values are mean ± SD or median [25 percentile, 75 percentiles] according to distribution.

ACE=angiotensin converting enzyme; ARB=angiotensin receptor blocker; BNP=brain natriuretic peptide; BMI=body mass index; CABG=coronary artery bypass graft; CAD=coronary artery disease; CCB=calcium channel blocker; CHF=congestive heart failure; CKD=chronic kidney disease; CVA=cerebrovascular accident; DBP=diastolic blood pressure; DM=diabetes mellitus; ESRD=end-stage renal disease; HbA1C=hemoglobin A1C; HDL-C=high-density lipoprotein cholesterol; HR=heart rate; LDL-C=low-density lipoprotein cholesterol; LVEF=left ventricular ejection fraction; MI=myocardial infarction; NOAC=new oral anticoagulant; NSTEMI=non-ST segment elevation myocardial infarction; PCI=percutaneous coronary intervention; STEMI= ST segment elevation myocardial infarction

Supplementary Table 2. Procedural profiles and in-hospital complications in STEMI and NSTEMI patients						
	**STEMI (N=256)**			**NSTEMI (N=293)**		
	Immediate (N=109)	Staged (N=147)	p	Immediate (N=220)	Staged (N=73)	p
STEMI						
DBT (min)	72.5 [55.0;98.5]	68.0 [54.0;83.0]	0.113	625.5 [219.0;1180.0]	350.0 [167.0;869.0]	0.001
Culprit vessel			0.002			0.009
LAD	55 (50.5%)	42 (28.6%)		78 (35.5%)	16 (21.9%)	
LCX	13 (11.9%)	22 (15.0%)		73 (33.2%)	20 (27.4%)	
RCA	41 (37.6%)	83 (56.5%)		69 (31.4%)	37 (50.7%)	
3 vessel disease	39 (35.8%)	71 (48.3%)	0.061	65 (29.5%)	36 (49.3%)	0.003
Left main disease	3 (2.8%)	7 (4.8%)	0.621	3 (1.4%)	4 (5.5%)	0.12
Culprit lesion No.	1.0 [1.0; 1.0]	1.0 [1.0; 1.0]	0.27	1.0 [1.0; 1.0]	1.0 [1.0; 1.0]	0.071

Non-culprit lesion No.	1.0 [1.0; 2.0]	1.0 [1.0; 2.0]	0.031	1.0 [1.0; 1.0]	1.0 [1.0; 2.0]	0.002
Transfemoral	13 (11.9%)	38 (25.9%)	0.009	19 (8.6%)	18 (24.7%)	0.001
Stent insertion for culprit lesion	108 (99.1%)	146 (99.3%)	1	214 (97.7%)	72 (98.6%)	0.367
Gp IIb IIIA inhibitor	32 (29.4%)	41 (27.9%)	0.907	18 (8.2%)	12 (16.4%)	0.073
Direct stenting for culprit artery	15 (13.8%)	11(7.5%)	0.151	22 (10.0%)	0 (0.0%)	0.011
Image guided PCI for culprit artery	16 (14.7%)	23 (15.6%)	0.97	66 (30.0%)	13 (17.8%)	0.06
Culprit total No. of stents	1.3 ± 0.6	1.2 ± 0.4	0.214	1.2 ± 0.5	1.3 ± 0.6	0.264
Culprit-mean stent size	3.1 ± 0.4	3.2 ± 0.5	0.299	3.1 ± 0.5	3.2 ± 0.4	0.332
Culprit-mean stent length	30.0 ± 7.2	28.9 ± 7.6	0.249	27.3 ± 7.0	29.1 ± 7.2	0.057
Culprit-total stent length	38.4 ± 20.2	34.6 ± 15.0	0.095	34.0 ± 15.6	38.8 ± 18.8	0.056
Culprit-procedural success	109 (100.0%)	147 (100.0%)	1	219 (99.5%)	73 (100.0%)	1
FFR guided	55 (50.5%)	72 (49.0%)	0.914	115 (52.3%)	37 (50.7%)	0.92

Any PCI for non-culprit artery(s)	95 (87.2%)	126 (85.7%)	0.882	170 (77.3%)	59 (80.8%)	0.637
Stent insertion for non-culprit	93 (97.9%)	118 (93.7%)	0.295	163 (95.9%)	57 (96.6%)	1
Image guided PCI for non-culprit[a]	22 (23.2%)	48 (38.1%)	0.027	44 (25.9%)	20 (33.9%)	0.311
Non-culprit maximal diameter stenosis (%)*	77.7 ± 11.9	77.9 ± 10.9	0.881	75.0 ± 11.0	75.7 ± 12.5	0.640
Non-culprit total lesion length (mm) *	20.4 ± 10.3	23.6 ± 12.1	0.024	22.3 ± 12.4	25.7 ± 12.0	0.031
Non-culprit FFR before PCI	0.70 ± 0.1	0.70 ± 0.1	0.470	0.70 ± 0.1	0.70 ± 0.1	0.569
Non-culprit total No. of stents	1.1 ± 0.6	1.3 ± 0.8	0.023	1.0 ± 0.8	1.4 ± 1.0	0.001
Non-culprit-mean stent size*	3.1 ± 0.5	3.0 ± 0.5	0.521	3.0 ± 0.4	3.0 ± 0.4	0.913
Non-culprit-total stent length*	32.3 ± 15.0	40.8 ± 20.9	0.001	36.2 ± 22.3	50.1 ± 20.9	<0.001
Non-culpirt-procedural success*	95 (100.0%)	126 (100.0%)		170 (100.0%)	59 (100.0%)	
Total No. of stents	2.3 ± 0.8	2.5 ± 1.0	0.289	2.2 ± 1.0	2.7 ± 1.2	0.001

Hospital stays	3.0 [2.0, 5.0]	3.0 [2.0, 5.0]	0.137	2.0 [1.0, 3.0]	2.0 [1.0, 5.0]	0.297
In-hospital complications						
Any complications	3 (2.8%)	8 (5.4%)	0.461	9 (4.1%)	6 (8.2%)	0.28
CHF	1 (0.9%)	0 (0.0%)	0.88	2 (0.9%)	1 (1.4%)	1
Emergent PCI	0 (0%)	0 (0%)		0 (0%)	0 (0%)	
Emergent CABG	0 (0%)	0 (0%)		0 (0%)	0 (0%)	
Cardiogenic shock	0 (0.0%)	5 (3.4%)	0.137	1 (0.5%)	1 (1.4%)	0.998
Contrast reaction	0 (0.0%)	0 (0.0%)	1	1 (0.5%)	0 (0.0%)	1
Cardiac tamponade	0 (0.0%)	0 (0.0%)		0 (0.0%)	0 (0.0%)	
Bleeding at access site	1 (0.9%)	2 (1.4%)	1	1 (0.5%)	0 (0.0%)	1
Access site occlusion	0 (0.0%)	0 (0.0%)	1	0 (0.0%)	1 (1.4%)	0.561
Access site dissection	0 (0.0%)	0 (0.0%)		0 (0.0%)	0 (0.0%)	
Access site AV fistula	0 (0.0%)	0 (0.0%)		0 (0.0%)	0 (0.0%)	

Peripheral embolization	0 (0.0%)	0 (0.0%)	1	0 (0.0%)	1 (1.4%)	0.561
Pseudoaneurysm	0 (0.0%)	0 (0.0%)		0 (0.0%)	0 (0.0%)	
Cardiac arrest	0 (0.0%)	1 (0.7%)	1	0 (0.0%)	1 (1.4%)	0.561
CIN	0 (0.0%)	0 (0.0%)	1	1 (0.5%)	1 (1.4%)	0.998

Values are mean ± SD or median [25 percentile, 75 percentiles] according to distribution.

*Patients who did not have PCI for non-culprit artery(s) were excluded.

AV=arteriovenous; CABG=coronary artery bypass graft; CHF=congestive heart failure; CIN=contrast induced nephropathy; DBT=door to balloon time; FFR=fractional flow reserve; LAD=left anterior descending; LCX=left circumflex artery; NSTEMI=non-ST segment elevation myocardial infarction; PCI=percutaneous coronary intervention; STEMI= ST segment elevation myocardial infarction

**Supplementary Table 3. Results of Cox Proportional Hazard Modelling**						
**AMI**						
	**Univariable HR of staged CR (95% CI)**	**P value**	**Multivariable HR of staged CR (95% CI)**	**P value**	**† Adjusted HR of staged CR (95% CI)**	**P value**
Composite outcome	1.03 (0.60-1.77)	0.905	0.97 (0.55-1.72)	0.928	0.81 (0.43-1.53)	0.528
All-cause death	1.05 (0.43-2.56)	0.904	1.18 (0.56-2.48)	0.66	1.01 (0.98-1.03)	0.46
Any AMI	0.76 (0.34-1.71)	0.522	0.74 (0.35-1.59)	0.45	1.01 (0.95-1.01)	0.28
Any RR	0.88 (0.39-1.99)	0.766	1.04 (0.92-1.18)	0.55	1.00 (0.96-1.02)	0.55
**STEMI**						
Composite outcome	0.84 (0.36-1.94)	0.676	1.08(0.48-2.45)	0.84	0.98 (0.93-1.03)	0.49
All-cause death	3.76 (0.41-33.95)	0.237	1.59(0.52-4.82)	0.41	1.01 (0.99-1.03)	0.17
Any AMI	0.500(0.14-1.77)	0.284	0.75(0.26-2.13)	0.6	0.98 (0.94-1.02)	0.34
Any RR	0.42(0.12-1.41)	0.163	0.62(0.21-1.85)	0.4	0.97 (0.93-1.01)	0.2

**NSTEMI**						
Composite outcome	1.52 (0.74-3.11)	0.255	1.00 (0.47-2.11)	0.99	0.99 (0.91-1.07)	0.84
All-cause death	1.11 (0.35-3.48)	0.849	1.01 (0.35-2.91)	0.98	1.00 (0.94-1.08)	0.8
Any AMI	1.31 (0.46-3.72)	0.613	1.07 (0.39-2.93)	0.89	0.97 (0.93-1.02)	0.37
Any RR	1.85 (0.61-5.56)	0.272	0.95 (0.31-2.09)	0.94	0.99 (0.94-1.04)	0.79

* Composite outcome = all-cause death + myocardial infarction (including periprocedural MI) + any repeated revascularization

† Overlap weighting adjusted multivariable analysis

AMI=acute myocardial infarction; HR=hazard ratio; MI=myocardial infarction; NSTEMI=non-ST segment elevation myocardial infarction; RR=repeated revascularization; STEMI=ST segment elevation myocardial infarction

AMI	Univariate	P value	Multivariate	P value	† Adjusted HR of staged CR (95% CI)	P value
Composite outcome	1.03 (0.60-1.77)	0.905	0.97 (0.55-1.72)	0.928	0.81 (0.43-1.53)	0.528
All-cause death	1.05 (0.43-2.56)	0.904	1.18 (0.56-2.48)	0.66	1.01 (0.98-1.03)	0.46
Any AMI	0.76 (0.34-1.71)	0.522	0.74 (0.35-1.59)	0.45	1.01 (0.95-1.01)	0.28

Any RR	0.88 (0.39-1.99)	0.766	1.04 (0.92-1.18)	0.55	1.00 (0.96-1.02)	0.55
STEMI						
Composite outcome	0.84 (0.36-1.94)	0.676	1.08(0.48-2.45)	0.84	0.98 (0.93-1.03)	0.49
All-cause death	3.76 (0.41-33.95)	0.237	1.59(0.52-4.82)	0.41	1.01 (0.99-1.03)	0.17
Any AMI	0.500(0.14-1.77)	0.284	0.75(0.26-2.13)	0.6	0.98 (0.94-1.02)	0.34
Any RR	0.42(0.12-1.41)	0.163	0.62(0.21-1.85)	0.4	0.97 (0.93-1.01)	0.2
NSTEMI						
Composite outcome	1.52 (0.74-3.11)	0.255	1.00 (0.47-2.11)	0.99	0.99 (0.91-1.07)	0.84
All-cause death	1.11 (0.35-3.48)	0.849	1.01 (0.35-2.91)	0.98	1.00 (0.94-1.08)	0.8
Any AMI	1.31 (0.46-3.72)	0.613	1.07 (0.39-2.93)	0.89	0.97 (0.93-1.02)	0.37
Any RR	1.85 (0.61-5.56)	0.272	0.95 (0.31-2.09)	0.94	0.99 (0.94-1.04)	0.79

Supplementary Table 4. Final models of OW-adjusted multivariable Cox proportional models for composite outcome

AMI		
	† Adjusted HR of staged CR (95% CI)	P value
Staged PCI	0.81 (0.43-1.53)	0.528
Age≥65	1.34 (0.65-2.59)	0.382
STEMI	0.56 (0.31-1.01)	0.054
FFR guidance	0.26 (0.14-0.51)	<0.001
Diabetes	1.95 (1.00-3.77)	0.047
CKD	1.11 (0.26-4.62)	0.879
LVEF	0.95 (0.92-0.98)	0.001
Left main disease	1.85 (0.36-9.43)	0.456
3-vessel disease	1.33 (0.74-2.39)	0.325
STEMI		
Staged PCI	0.98 (0.93-1.03)	0.49
Age≥65	1.02 (0.97-1.08)	0.36
FFR guidance	0.95(0.91-1.00)	0.1
Diabetes	1.04 (0.97-1.10)	0.2
LVEF	0.99 (0.992-0.999)	0.011
3-vessel disease	1.01 (0.96-1.06)	0.58
NSTMI		

Staged PCI	0.99 (0.91-1.07)	0.84
Age≥65	1.06 (1.00-1.13)	0.45
FFR guidance	0.90 (0.84-0.96)	0.001
Diabetes	1.06 (0.99-1.14)	0.058
CKD	1.12 (0.93-1.36)	0.22
LVEF	0.99 (0.99-1.00)	0.059
3-vessel disease	1.03 (0.96-1.10)	0.34

AMI=acute myocardial infarction; CI=confidence interval; CKD=chronic kidney disease; FFR=fractional flow reserve; HR=hazard ratio; LVEF=left ventricular ejection fraction; NSTEMI=non-ST segment elevation myocardial infarction; OW=overlap weighting; PCI=percutaneous coronary intervention; STEMI=ST segment elevation myocardial infarction

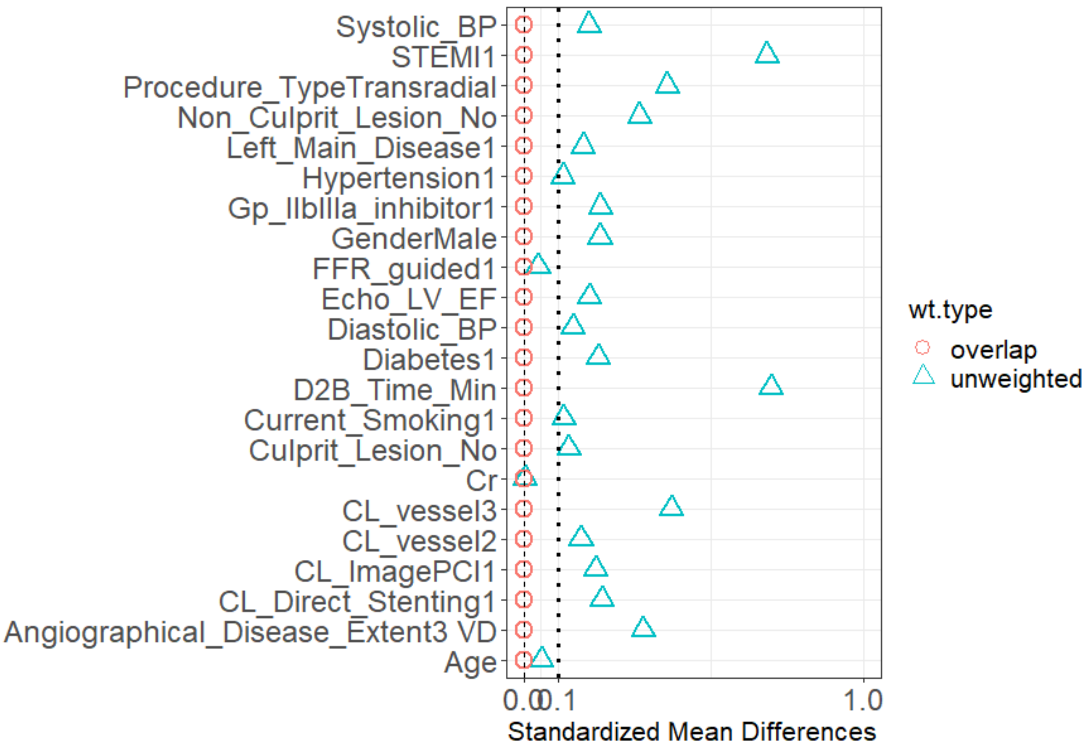

Supplementary Figure 1. Balancing before and after weighting in all acute myocardial infarction (AMI).

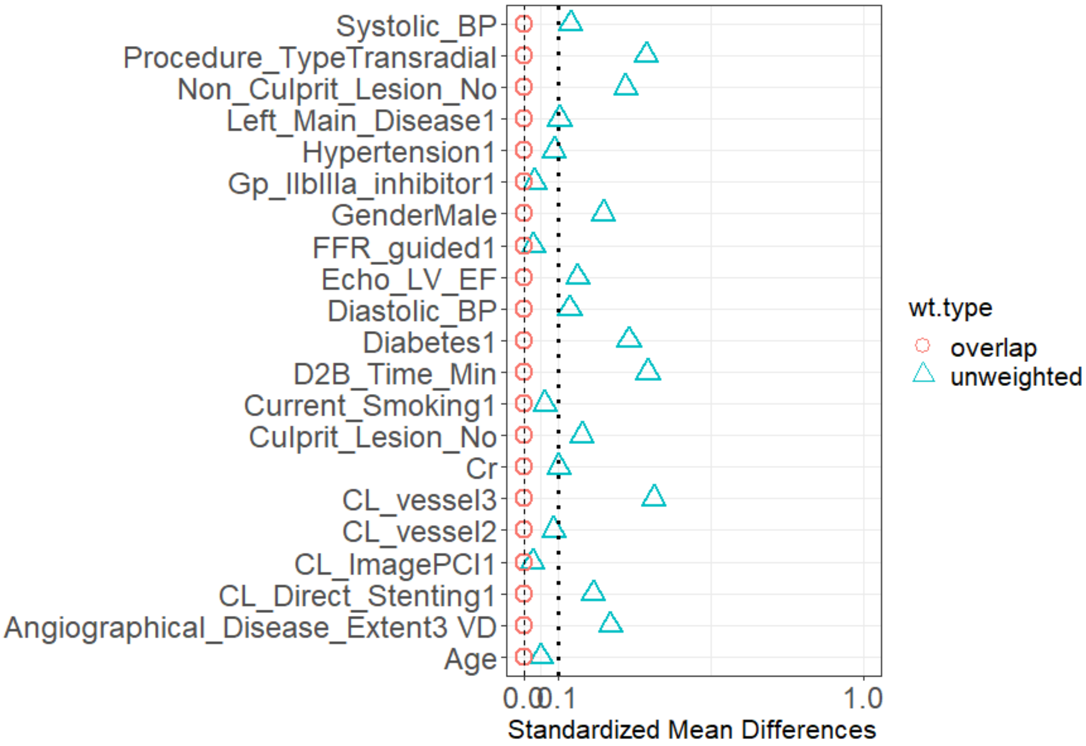

Supplementary Figure 2. Balancing before and after weighting in ST segment elevation myocardial infarction (STEMI).

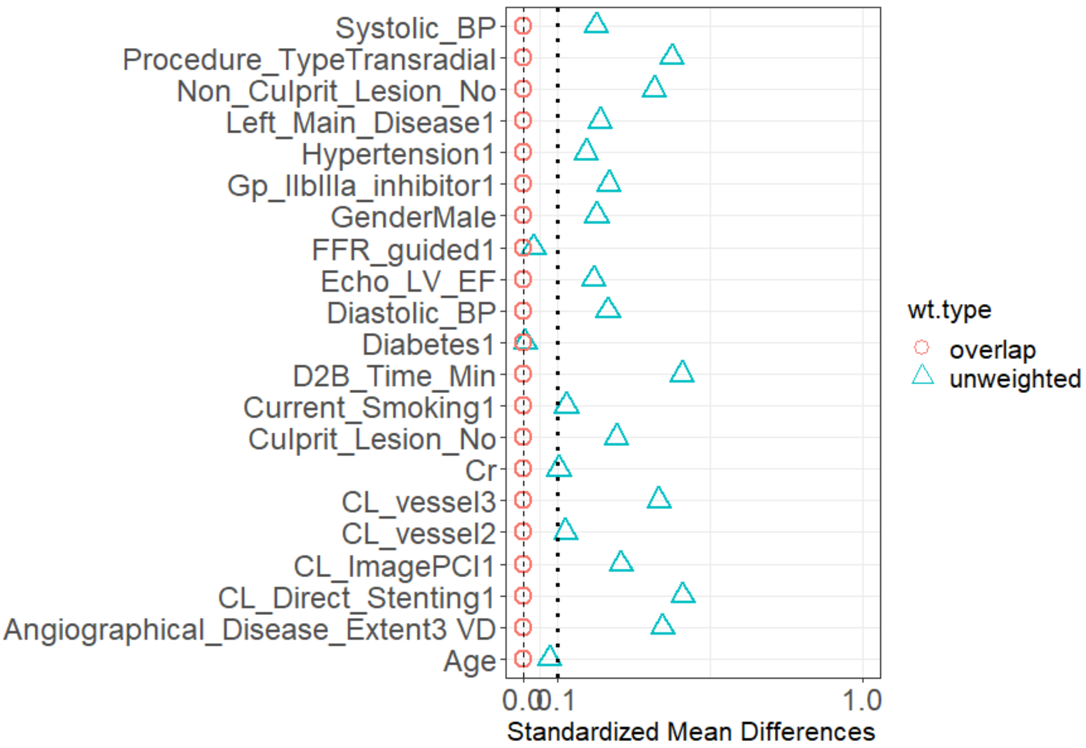

Supplementary Figure 3. Balancing before and after weighting in non-ST-segment elevation myocardial infarction (NSTEMI)

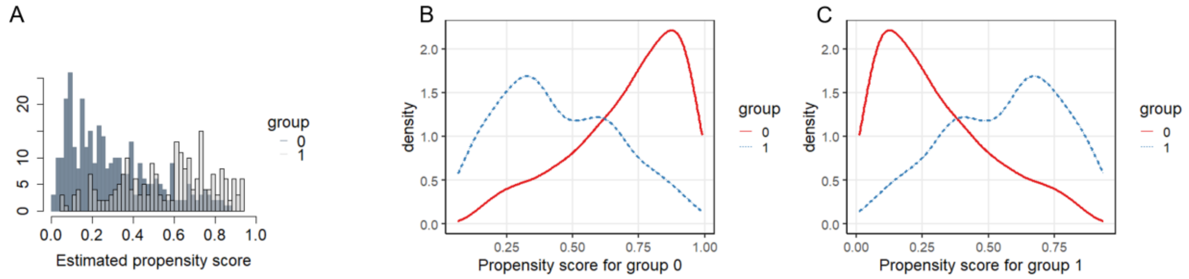

Supplementary Figure 4. Histogram for propensity score (A), and density plot describing overlap weighting in all patients (B, C)

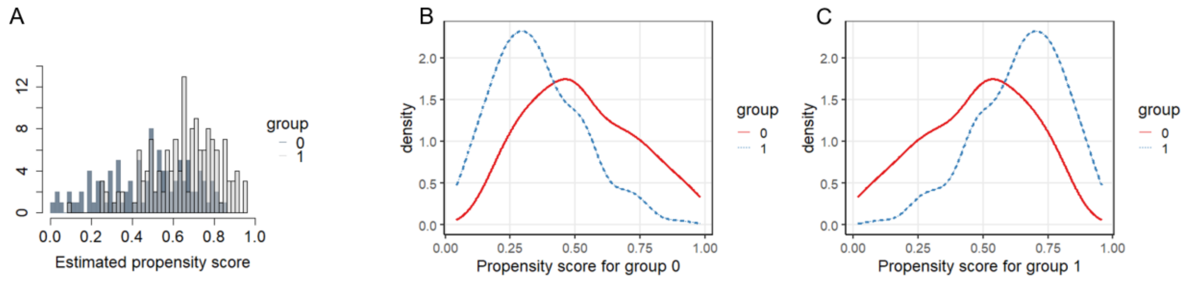

Supplementary Figure 5. Histogram for propensity score (A), and density plot describing overlap weighting in patients with ST-segment elevation myocardial infarction (B, C)

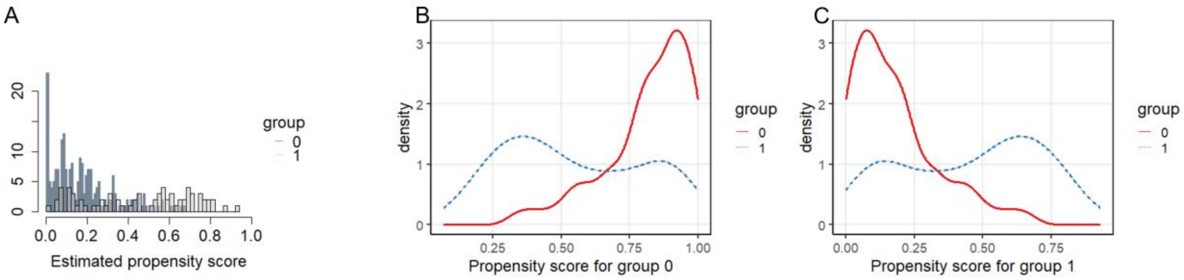

Supplementary Figure 6. Histogram for propensity score (A), and density plot describing overlap weighting in patients with non-ST-segment elevation myocardial infarction (B, C)

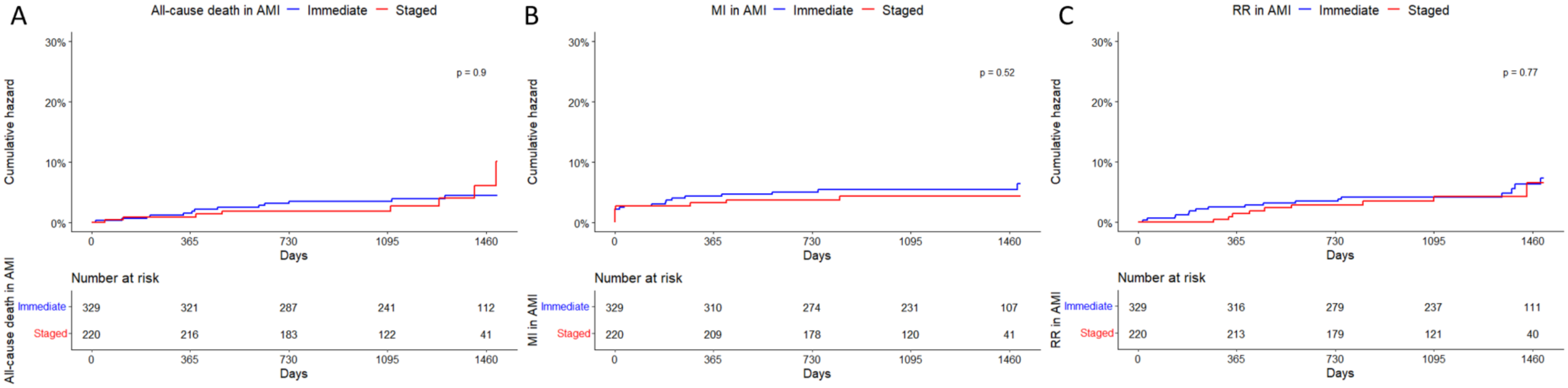

Supplementary Figure 7. Kaplan-Meier curve of all-cause death (A), MI (B), and repeated revascularization (C) in patients with AMI with multivessel disease treated with immediate or staged PCI strategy for non-culprit artery(s).

AMI=acute myocardial infarction; CI= confidence interval; HR= hazard ratio; MI=myocardial infarction; POCO=patient-oriented composite outcome; RR=repeated revascularization

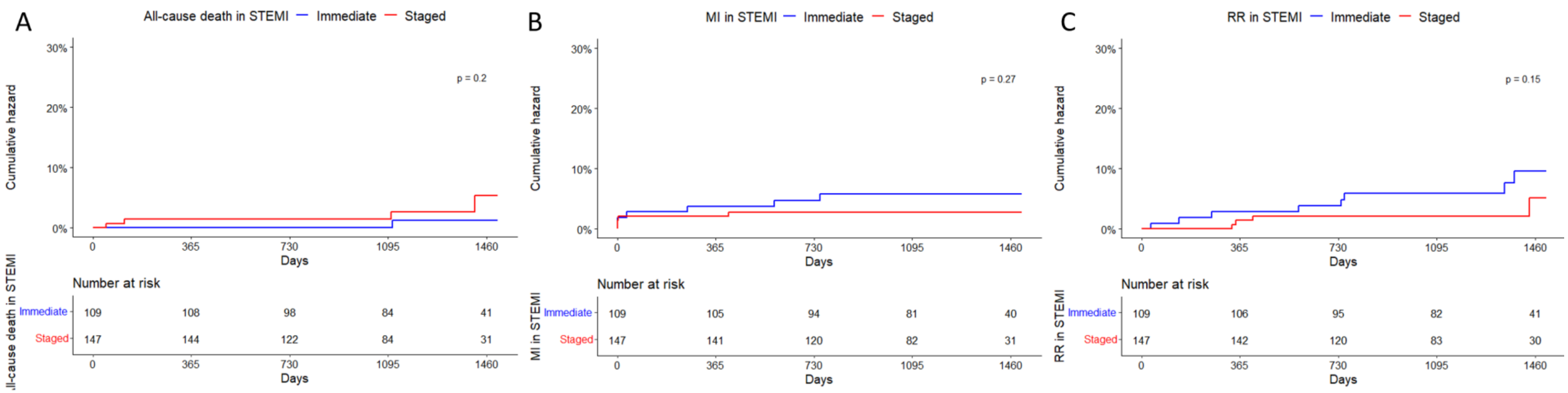

Supplementary Figure 8. Kaplan-Meier curve of all-cause death (A), MI (B), and repeated revascularization (C) in patients with STEMI with multivessel disease treated with immediate or staged PCI strategy for non-culprit artery(s).

AMI=acute myocardial infarction; CI= confidence interval; HR= hazard ratio; MI=myocardial infarction; POCO=patient-oriented composite outcome; RR=repeated revascularization; STEMI= ST segment elevation myocardial infarction

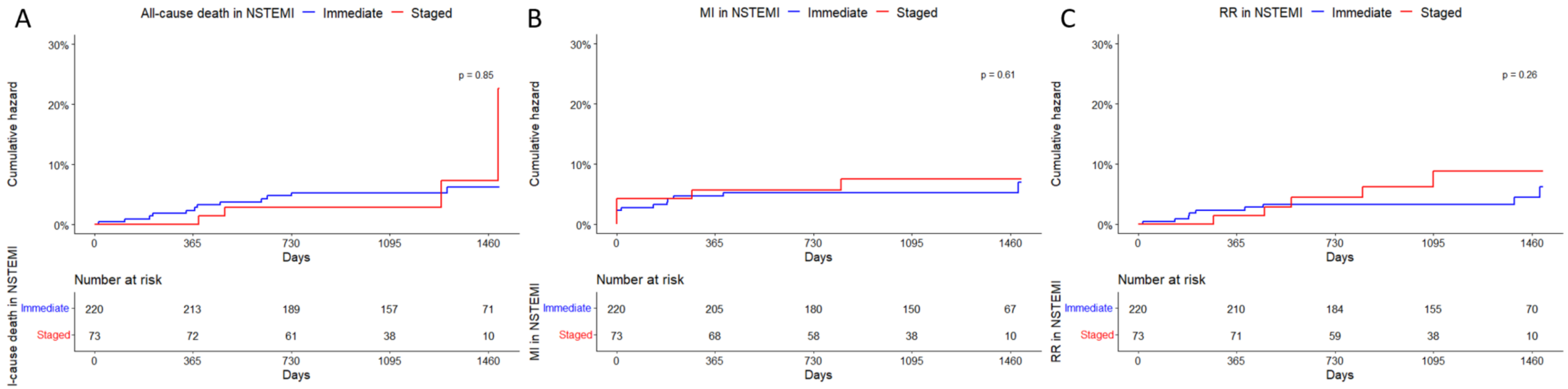

Supplementary Figure 9. Kaplan-Meier curve of all-cause death (A), MI (B), and repeated revascularization (C) in patients with NSTEMI with multivessel disease treated with immediate or staged PCI strategy for non-culprit artery(s).

AMI=acute myocardial infarction; CI= confidence interval; HR= hazard ratio; MI=myocardial infarction; NSTEMI= non-ST segment elevation myocardial infarction; POCO=patient-oriented composite outcome; RR=repeated revascularization.
